# Green Villages, the Pandemic, and the Future of California Urbanism

**DOI:** 10.3390/ijerph21121591

**Published:** 2024-11-29

**Authors:** René Davids

**Affiliations:** College of Environmental Design, University of California, Berkeley, CA 94720, USA; rdavids@berkeley.edu

**Keywords:** multi-family housing, COVID-19 pandemic, case studies, California, natural environments, urban agriculture communal spaces, social interaction

## Abstract

During the COVID-19 pandemic, the role of housing in controlling the spread of the virus was limited, as policies primarily focused on short-term measures such as lockdowns and social distancing. As the pandemic recedes, a shift has occurred towards restructuring the environment to confront future health crises better. This research thoroughly evaluates existing literature and housing complexes. It recommends that future projects prioritize several key features: ample exposure to natural environments, opportunities for growing food, encouragement of casual social interactions, inclusion of communal spaces, and provision of areas for exercise to help reduce the risks of contagion and alleviate the mental health impacts on residents. Based on research conducted during and after the pandemic, current recommendations for housing often provide generalized suggestions or propose ideal layouts through diagrams. This approach can be unrealistic from both spatial and economic perspectives and fails to inspire or stimulate creativity. This paper, by contrast, reviews and analyzes historical housing projects while critically examining three case studies that have the potential to inspire future designs. The goal is to provide officials, architects, and stakeholders with a series of practical possibilities and guidelines that contribute to the post-COVID home design process by making it more health-conscious and fostering the creation of new types of neighborhoods that can significantly impact the planning of cities in California.

## 1. Introduction

Having first emerged in Wuhan, China, in December 2019 and then spreading rapidly throughout the world, the COVID-19 pandemic triggered a crisis like none other recalled in modern times. Many countries instigated lockdowns that brought their economies to a standstill. Many lives were affected in various ways due to this crisis: economic slowdowns, job losses, the rise of technology, automation causing further job losses, an increase in digital currencies, lower returns on savings, greater inequalities, and rising debts. The situation revived interest among design professionals in the interrelationships of architecture, urban planning, and health with an intensity not seen since pandemics marked the urban landscape during the nineteenth and early mid-twentieth centuries. Throughout the cholera outbreaks in the XIX century, many parts of cities were redesigned to provide clean air and water. In Paris, 12,000 buildings were torn down and replaced with tree-lined boulevards and parks to bring fresh air and light into the city grid. In London, the scenic promenades and gardens of the Victoria Embankment resulted from a new sewerage system to prevent infections. Frederick Law Olmsted, who tragically lost his first son to cholera, advocated for the creation of Central Park in New York as the “lungs of the city”. In Philadelphia, development along the Schuylkill River was removed and replaced with Fairmount Park to safeguard the water supply [1]. Before the invention of antibiotics, access to open space, sunlight, and fresh air was considered the most effective response to a deadly disease.

In the early 20th century, epidemics of tuberculosis, cholera, and influenza prompted the development of international modernism, particularly in the design of functional multifamily housing. These buildings were constructed on pilotis and situated in park-like environments to allow unobstructed airflow underneath and optimize exposure to sunlight and wind. After the mid-twentieth century, architects increasingly overlooked public health considerations in their designs. This neglect resulted in significant avoidable challenges for people living in many residential buildings constructed during the twentieth century, especially during the COVID-19 pandemic.

The health emergency profoundly impacted California. The hardest-hit neighborhoods had three times the rate of overcrowded homes and twice the rate of poverty compared to neighborhoods that largely escaped the virus. People of color disproportionately populated the areas with the most infections. About 6.3 million Californians, or 16%, live in overcrowded housing, with one-third severely overcrowded. This situation involved suburban houses intended for one family inhabited instead by multiple families or multifamily complexes built without consideration for health emergencies. California has the second-highest rate of crowded households nationwide, more than double the nationwide rate.

About two-thirds of the individuals living in overcrowded homes—roughly 4 million—are essential workers, meaning they work in health care, energy, and emergency services or reside with at least one essential worker. Social distancing is particularly challenging for these workers, as they must leave their homes regularly to help keep the rest of the U.S. fed and sheltered. Over one-third of California’s labor force is employed in jobs requiring physical presence, such as farming, fishing, or forestry. Almost a third of farm workers and restaurant workers live in overcrowded homes.

The generally accepted definition of overcrowding is a household with more than one person per room—including bedrooms, kitchens, and living rooms, excluding bathrooms. Severely overcrowded households have more than 1.5 people per room. The Public Policy Institute of California (PPIC) found that 16% of essential workers live in overcrowded housing, compared to 12% of nonessential workers [2,3].

Preparedness and prevention are critical for safeguarding public health and ensuring societal and economic resilience against infectious disease outbreaks. While strengthening healthcare infrastructure and enhancing the public health workforce are essential components of preparedness, housing design also plays a crucial role in developing neighborhoods equipped to confront the next pandemic.

## 2. Literature Background

The World Health Organization has provided evidence that the COVID-19 virus primarily spreads through close contact between individuals, typically at a conversational distance. It can be transmitted when an infected person coughs, sneezes, speaks, sings, or breathes, releasing small liquid particles from their mouth or nose. Additionally, the virus can spread in poorly ventilated or crowded indoor settings where people are close for extended periods. In these environments, aerosols can linger in the air and travel beyond the usual conversational distance [4,5]. Spatial conditions and usage can significantly influence the likelihood of virus transmission.

This literature review identifies and evaluates relevant peer-reviewed studies on COVID-19 and housing. It encompasses multiple disciplines, including architectural design, environmental psychology, building science, and engineering. While examining the factors contributing to increased infection risks and mental health issues, it also explores spatial relationships, modifications, and potential improvements to prevent or reduce the risk of future infections.

Researchers recommend adopting sustainable technologies to address energy and water consumption during apandemic and reduce the risk of viral transmission. They advocate for the broader implementation of touchless and automatic systems and for using finishing materials with low viral survivability. The literature suggests enhancing communication technologies through remote services and automatic systems to improve safety and reduce transmission risks. These improvements can help regulate comfort parameters such as air quality, lighting, temperature, and humidity more effectively, but these conditions do not by definition apply to the homeless. Different modes of urban mobility and transportation technologies can also help mitigate infection risks [1,6,7,8,9].

Numerous research articles and general observations on mental health issues during the pandemic highlight the significance of fostering a strong connection between homes and green spaces. In response, many individuals desired to move to the suburbs, believing that being close to green spaces would positively impact their mental and physical well-being. They sought neighborhoods with lower population densities, detached houses, and spacious yards. Some even argued that living in the suburbs in the U.S. served as a form of social distancing, as it entails residing in detached, single-family homes and commuting alone by car, which could help reduce the spread of COVID-19 [10]. Evidence indicates that the lockdown experience during the pandemic’s peak was significantly worse for people with no outdoor space than those with gardens or access to fresh air [11]. A survey in late 2020 revealed that 86 percent of people wanted to spend more time in parks and squares than before the pandemic. This desire was explained by the prolonged isolation, stress, grief, and the inability to visit with loved ones indoors due to limited mobility during the implementation of social distancing policies. The adverse psychological effects of quarantines included post-traumatic stress symptoms, confusion, and anger. Stressors included longer quarantine duration, infection fears, frustration, boredom, inadequate supplies and information, financial loss, and stigma [11,12].

Green spaces provide settings for relaxation; enable the building of social connections outdoors; reduce depression, anxiety, and stress; allow for physical activity; stimulate a feeling of being closer to nature and resident wildlife; and contribute to increased happiness and life satisfaction across the lifespan [12,13]. The full range of functions that green spaces provide includes olfactory tactile and multisensory effects, such as, for example, the connection of perceived sound reduction due to vegetative screening of an adjacent noise source. Green spaces should not be regarded as positive assets for a community without considering their quality. These spaces vary in size and shape and encompass living and non-living environmental components, including vegetation density and different surface levels such as ground-level, elevated, and vertical spaces. Negative features, such as being near a polluted freeway or lacking accessibility, can deter people from visiting these areas and may diminish their health benefits. This is especially important for children and older adults, who are particularly vulnerable to air and noise pollution [14,15]. The relationship between homes and green spaces includes features such as windows that provide a direct visual link to nature, which can benefit our mental health. Additionally, it is recommended that we grow our food to reduce the risk of deprivation due to mobility restrictions imposed during the pandemic. Poor nutrition can lead to obesity, which increases the risk of severe illness from COVID-19 and may triple the likelihood of hospitalization due to impaired immune function.

Urban agriculture can take various forms, including community gardens, rooftop farms, hydroponic systems, and aeroponic and/or aquaponics vertical farming set-ups. Unlike individual home gardens, urban agriculture fosters social interactions and emotional support, promoting connections among people. Thus, community gardens can significantly build resilience in urban areas, helping to maintain or quickly restore essential functions during disruptions such as the pandemic [5,16,17,18,19,20,21,22,23].

Farming’s multifaceted role as a vehicle for social support and a way to provide fresh food to the community also applied to residences during the pandemic, which performed many functions beyond being mere dwellings. Throughout enforced lockdowns, homes transformed into spaces where individuals could perform work, study, exercise, and find solace.

Therefore, after the COVID-19 pandemic, architects, homeowners, and stakeholders must enhance residential buildings and ensure that their houses can withstand potential future pandemics and lockdowns. By doing so, they can ensure that their homes remain safe, comfortable, and functional in adverse and unpredictable health emergency conditions.

The COVID-19 pandemic has revealed a socio-economic divide across the country, illustrated by people’s varying abilities to minimize their exposure risk. For instance, some individuals could work from home [24], avoid sending their children to school, use private instead of public transport, live in single-family homes rather than public housing blocks, and afford home delivery services rather than shopping in person. This socio-economic disparity also impacts people’s ability to purchase custom-built homes or retrofit existing buildings.

Thus, there is a pressing need to design affordable multifamily complexes that reduce infection risk and alleviate anxieties triggered by the pandemic to cater to individuals with modest incomes [25]. These developments should incorporate spatial solutions that are affordable, replicable, and effective in preventing the public health disadvantages experienced during the COVID-19 pandemic.

Research emphasizes the need for home design flexibility to accommodate families’ diverse needs, especially during health-related challenging times. This flexibility can involve creating designated areas for offices, entertainment, exercise, or even extra bedrooms. Installing adjustable walls and screens or dividing open areas into smaller, functional spaces, such as a home office or an entertainment zone, can facilitate this adaptability. However, movable partitions have historically often been shown to be ineffective. A layout featuring smaller rooms connected to a larger space allows for private areas within the home. Additionally, incorporating a designated entry space can help minimize the risk of virus transmission during home deliveries, specifically designed to reduce the accidental introduction of pathogens into the home [19,20,26,27].

Several published studies analyze how the designed environment can help diminish the risks of COVID-19 transmission [5,19,20,21,26,27,28,29,30,31,32]. The proposals, however, are offered as ideas or spatial diagrams that avoid considering economic issues, skip the difficulties that might arise through design practice implementation, and play no role in inspiring new approaches.

Researcher D. Spenneman argues for creating a containment space that separates the largely uncontrollable external environment from a threat-reduced residential area in suburban homes, for example. This involves establishing distinct areas for entertaining visitors and private sleeping quarters and designing a spatially separated master bedroom as a self-isolation space [33].

These recommendations are presented with schematic drawings that could be helpful if the house was inhabited by one family, but they would provide limited benefits if the house is overcrowded, as the primary issue is the number of people rather than the layout of the space. Furthermore, implementing these suggestions would come with expenses related to increasing the unit’s square footage, which have not been considered.

Case studies, as opposed to diagrams, are built examples able to demonstrate the physical and economic viability of an idea. Thus, they play a crucial role in disseminating practical knowledge within the design field. Moreover, case studies can elevate recommendations beyond simple lists of pragmatic suggestions, fostering creativity and imagination.

This review analyzes three selected multi-family housing schemes to evaluate the design features that can create favorable conditions for residents during future health emergencies, including pandemics. By highlighting the positive aspects of these features, we can draw lessons from the COVID-19 pandemic to develop new technologies and design strategies that prepare for and mitigate the spread of future pandemics.

## 3. History of California Suburban Multi-Family Housing: Bungalow Courts and Courtyard Housing

Although multifamily complexes have long existed in suburban areas, they are not typically associated with them. Despite a prevalent perception of homogeneity, a carpet of single-family homes, nearly half of California’s new housing since 2010 has been in multi-unit buildings, with 85% of these being large structures of five or more units. This represents a significant shift from 2000 to 2010, where only one in four new units was part of a multi-unit building [13,34].

In California, the design of many high-density multi-family housing developments has been greatly influenced by the population’s preference for individual bungalows set in landscaped grounds. In the 1910s, as the population increased in Southern California neighborhoods, a new type of housing known as “courts” emerged along streetcar lines. These courtyard developments consisted of small bungalows arranged in pairs around a central courtyard, with parking typically located behind or to the side of the complex.

**Figure 1:**
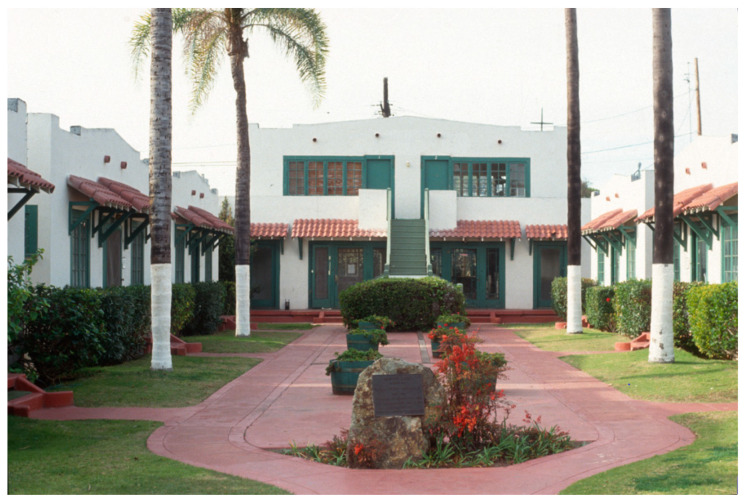
Bungalow court San Diego (photo by author 2004).

This type of housing required no more land than was previously allocated for two standard single-family bungalows while aiming to provide similar amenities. Bungalow courts represented higher-density complexes that were seamlessly integrated into single-family neighborhoods. Having a court adjacent to a single-family house indicated a subtle yet significant increase in density, often by four to six times (see Figure 1).

Bungalow courts were multi-family housing models that emphasized communal spaces alongside individual homes. They allowed residents to enjoy shared gardens and green alleys, which fostered social interaction in a suburban environment. However, with rising land costs and stringent parking requirements, these multi-family arrangements became increasingly expensive. In response to this challenge, a new type of building known as “courtyard housing” or “courts” emerged.

**Figure 2:**
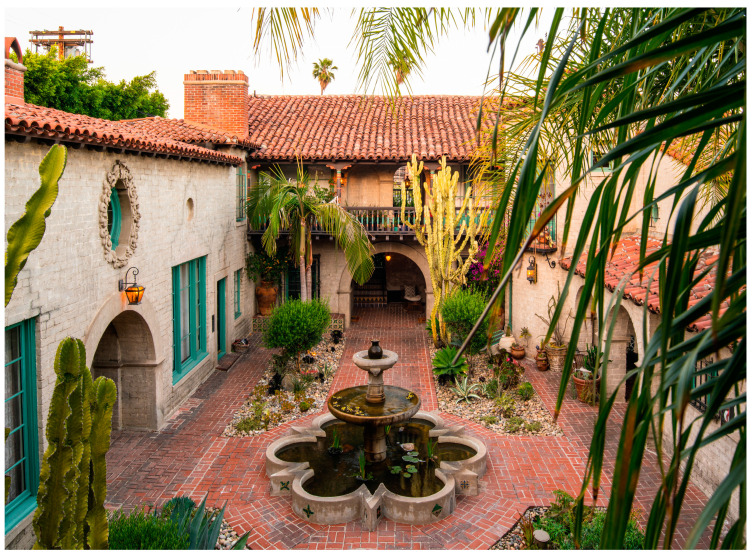
El Cabrillo Garden Court, Los Angeles (example of courtyard housing) Arthur and Nina Zwebell 1927 (photo courtesy Jordan Ashley Brett 2021).

Courtyard housing resembled a large villa with a central courtyard surrounded by apartments, creating a unified surface and a communal area for all residents. While bungalow courts were typically simple and modest, courtyard housing was more ornate, often featuring fountains and tiled surfaces inspired primarily by Spanish prototypes. These structures included spacious patios, fountains, verandas, and balconies that opened into the central courtyard (see Figure 2).

Additionally, courtyard housing often featured lush landscaping, providing serene spaces for relaxation and meditation, and their outdoor hallways and corridors promoted interaction among residents. Its focus on dedicated common outdoor spaces makes this housing a significant alternative to the idealized American dream of owning a stand-alone home [35].

## 4. Garden Apartments

Courtyard housing usually occupies large parcels of land, while the superblock planning concept from the Garden City movement, typically made up of multiple city blocks, influenced the so-called garden apartments in California. This movement, initiated by Ebenezer Howard during England’s Victorian era, aimed to create self-sustaining communities surrounded by green spaces in response to urban overcrowding and pollution.

American planners Clarence Stein and Henry Wright adapted Howard’s ideas on the East Coast, notably in New Jersey in 1929, where superblocks separated automobiles from pedestrians and included shared gardens.

In the early 1930s, Southern California faced a housing crisis due to a population boom. In 1935, the first self-contained garden apartment community, inspired by the Radburn plan, within Fair Lawn, New Jersey, typified by the home’s backyards facing the street and the fronts facing one another over common yards, was created. Collaborating architects, including R.D. Johnson and landscape architect Fred Barlow Jr., worked on the project, later renamed “Baldwin Hills Village”. The community featured residential buildings sharing walls with adjacent properties, oriented toward a central village green, occupying only 14 percent of the site. Construction began in 1941, and the apartments offered spacious layouts with large windows, private patios, and wood-burning fireplaces [16,36,37,38,39,40]. The garden apartments provided a sense of community and no responsibility for garden maintenance. The layout resonated with European social housing experiments of the 1920s when municipal planners and architects designed blocks of unadorned, minimalist, low-rise garden apartments and arranged them in rectilinear rows with plenty of green space in between [39,41]. This type of superblock planning—introduced to the United States by housing reformers like Catherine Bauer and implemented by regional planning exponents—aimed to provide a public or nonprofit sector solution to poor conditions in tenement blocks [42].

**Figure 3:**
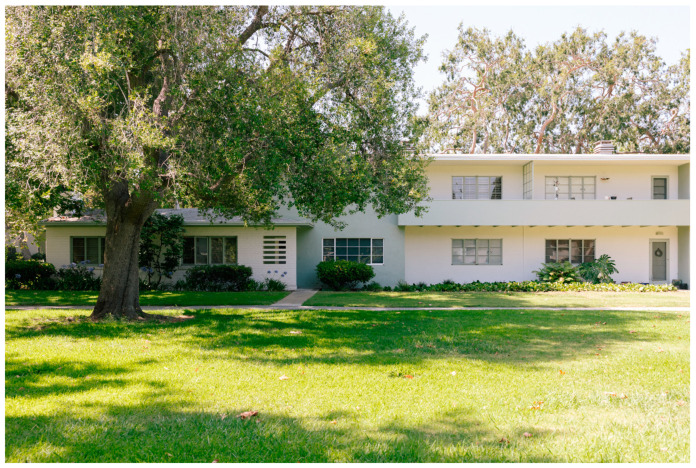
Baldwyn Hills Village. Photograph copyright © 2019 Liz Kuball (www.lizkuball.com).

The garden apartments were designed to create distinct semi-self-sufficient areas that foster social interaction and leisure opportunities. In the Baldwyn Hills Village development (Figure 3), the architects included a variety of recreational facilities for residents. These features included a clubhouse with a lending library, ping-pong tables, a darkroom, and a reading lounge with a fireplace and patio. A spacious area was also available for dances, church services, and community meetings. Nearby, a large playground was next to the clubhouse, while tennis courts were on either side of the Administration Building. Garden apartments have contributed to increased density in California cities while preserving a suburban character, helping to counteract the negative perceptions often associated with multifamily housing in the United States [40,43,44].

## 5. Bungalow Court Re-Interpretation: Daybreak Grove

Daybreak Grove is Escondido’s first multi-family housing development, designed to demonstrate that low-income housing can be high quality. Developed by the North County Housing Foundation and designed by Davids-Killory Architects, it features 13 units on less than an acre. The units are primarily intended for households led by mothers with children.

**Figure 4:**
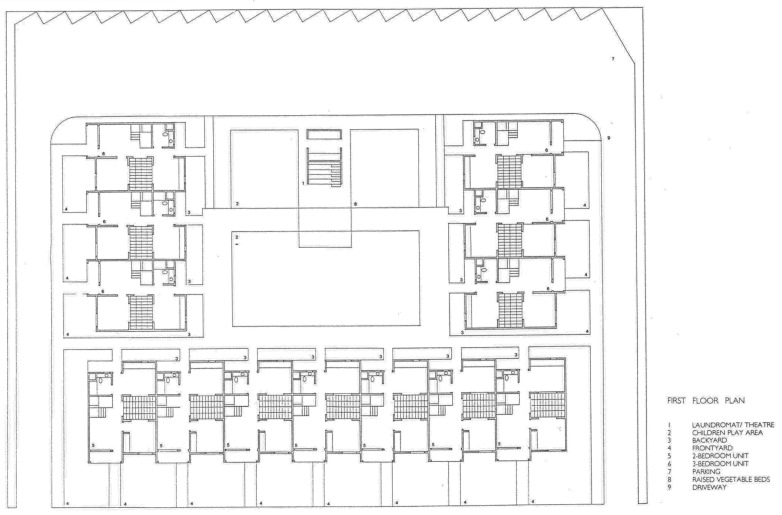
Daybreak Grove plan of the complex (drawing courtesy of Davids-Killory).

**Figure 5:**
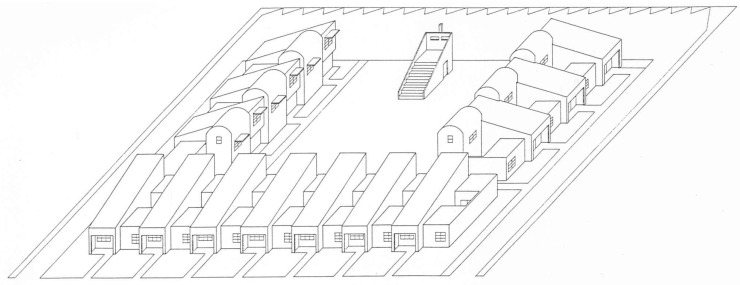
Daybreak Grove isometric of the complex (drawing courtesy of Davids-Killory).

**Figure 6:**
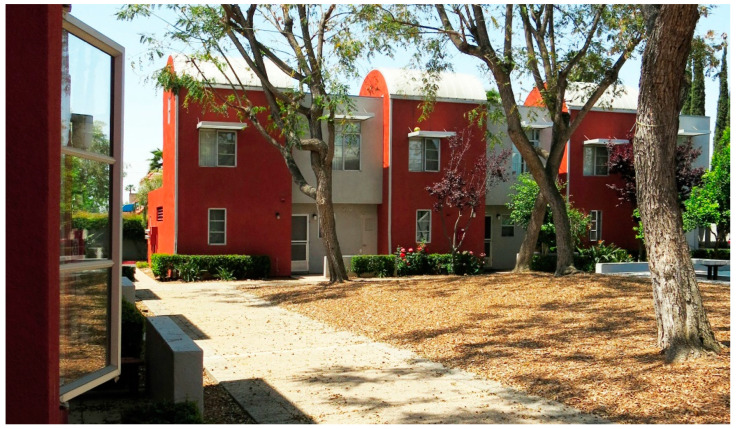
Daybreak Grove, courtyard (photo by author 2013).

California bungalow courts inspire the design, consisting of small houses with front and backyards centered around a communal courtyard (see Figure 4, Figure 5 and Figure 6). This courtyard includes picnic tables, a barbecue area, and a raised garden bed where residents can garden. The layout fosters a sense of community and encourages positive social interactions, and it can remain safe even during health emergencies.

Daybreak’s townhouses surround a shared courtyard, eliminating the need for communal lobbies, elevators, stairways, corridors, and HVAC systems, minimizing the potential for virus transmission through shared airspaces [45]. A laundromat is located beneath a community theater in the courtyard’s center. This setup is especially beneficial during pandemic lockdowns, as it helps alleviate feelings of isolation and provides opportunities for outdoor classes when schools are closed. The compact size of the project is advantageous, as research suggests that larger, densely populated buildings can negatively impact residents’ mental health due to increased social interaction, which can lead to stress.

The courtyard features Tipuana tipu trees, offering shade and beauty. Each unit’s rear patios and porches are protected by low walls, which serve as informal seating areas (see Figure 4 and Figure 5). These porches can be utilized as outdoor workspaces or offices during a health emergency unless it rains (see Table 1).

The building structures step back and forth, creating recessed entryways for added privacy. Their forms are highlighted by the pigmented stucco’s contrasting deep rust red and gray. The design includes seven units, each measuring 730 square feet, with alternating flat and shed roofs that create a lower profile on the south side of the courtyard facing the street.

**Figure 7:**
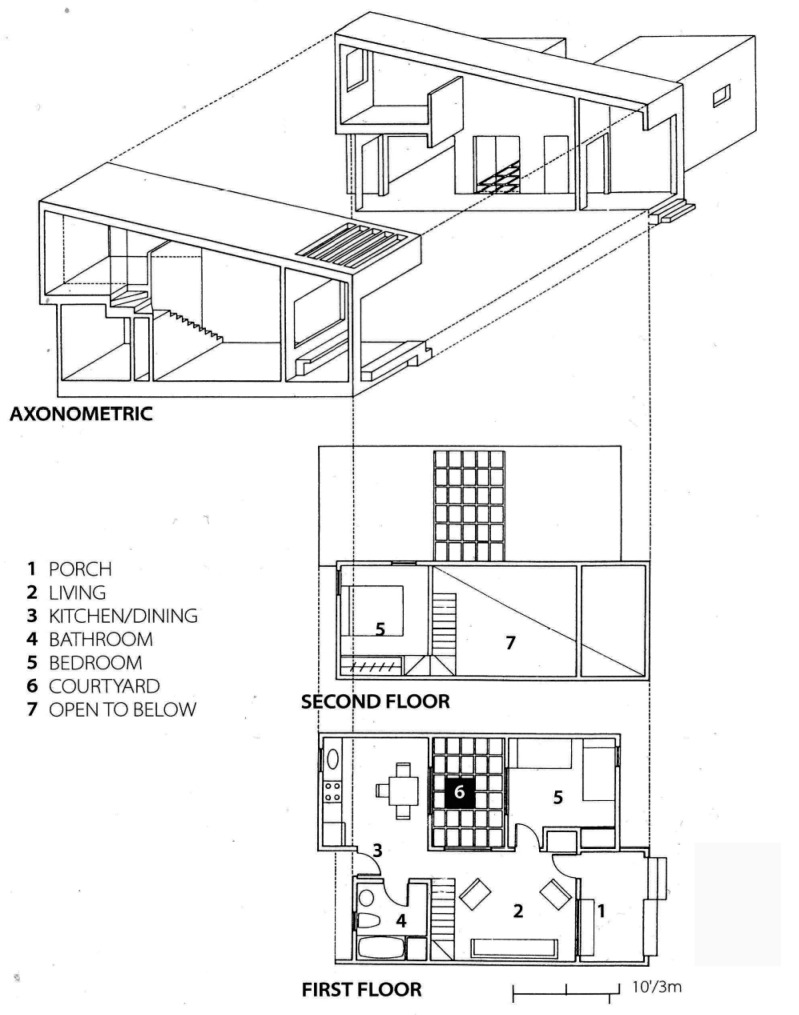
Daybreak Grove isometric of the complex (drawing courtesy of Davids-Killory).

The entry sequence for the units smoothly transitions from public to semi-private and private spaces, starting at the sidewalk and moving through the front yard, porch, and entrance. A bedroom located next to the entrance can serve as an office if the bed in that room is part of a bunk-bed arrangement in another bedroom. Each apartment features an internal courtyard that provides essential cross ventilation, which is vital for maintaining a healthy indoor climate. Operable windows in living and cooking spaces enhance indoor air quality and thermal comfort, promoting occupants’ well-being, productivity, and health within the unit (see Figure 7).

The adaptable layout of the units is crucial for supporting the evolving role of the home during health emergencies. The organization of the Daybreak units offers flexibility through the availability of porches, the shared courtyard, and the theater structure.

Functional flexibility at Daybreak Grove is achieved economically without increasing floor square footage or installing unique mechanisms, such as expensive moving wall systems that often fail due to mechanical failure or require time-consuming effort to move furniture.

## 6. Townhomes Re-Interpretation: Garden Village

Completed in 2016 in Berkeley, CA., the “Garden Village” housing project was designed by Stanley Saitowitz of Natoma Architects with Nautilus Group, a fully integrated real estate investment, development, and design-build construction group, as the architect of record. The development was made partly possible by a Berkeley City Council initiative that approved an ordinance eliminating parking requirements for residential development that had previously mandated one off-street parking space for every new residential unit. Instead of parking spots for residents, Garden Village offers bike parking, discounted transit tickets, and on-site car-sharing services [46].

**Figure 8:**
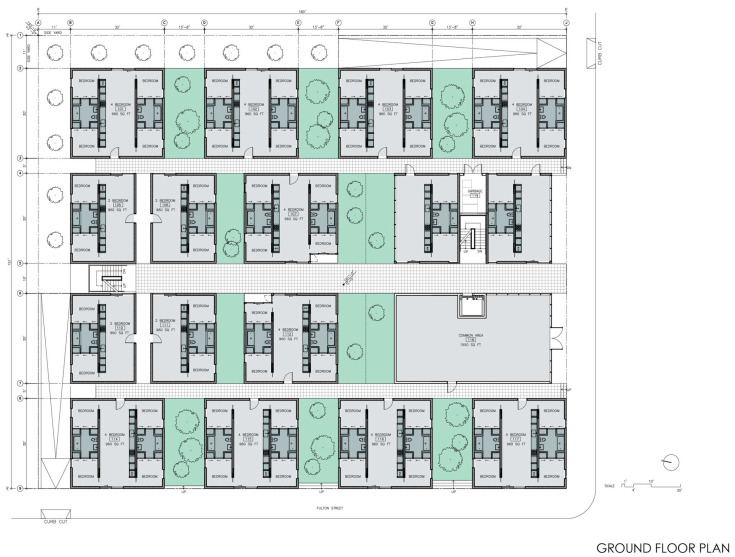
Garden Village ground floorplan (courtesy Natoma Architects Inc., San Francisco, CA, USA).

**Figure 9:**
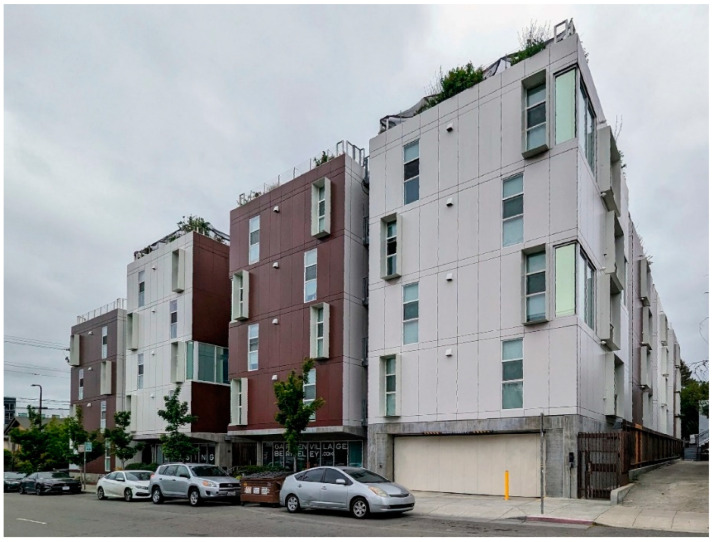
Garden Village façade (photo by author 2023).

**Figure 10:**
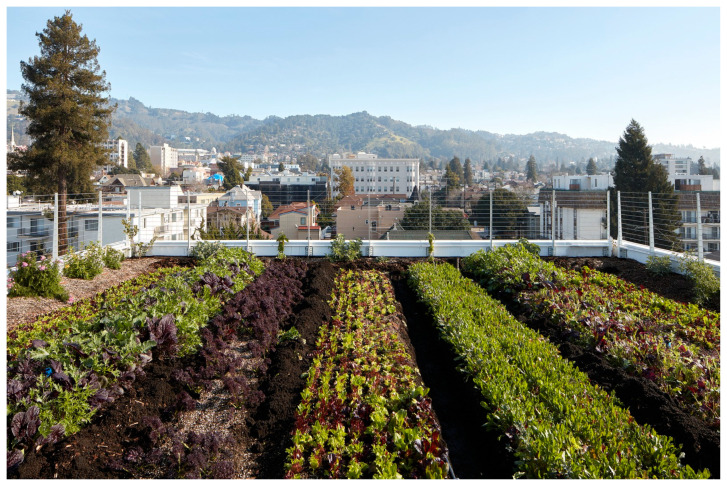
Garden Village Roof Farm (photo by author 2023).

The circulation consists of a well-ventilated double-loaded corridor that reduces the risk of contagion and a single elevator.(see Figure 8). More elevators would diminished health risks but increased construction cost. Reminiscent of narrow, vertical townhomes like the famous “Postcard Row” at Alamo Square in San Francisco, the detached towers of Garden Village allow every bedroom and living room access to air and light, providing an exceptionally well-ventilated and healthy environment for an apartment complex, a great benefit during health emergencies (see Figure 9). The closeness of one of the bedrooms to the entrance allows it to comfortably become an office. In the four-bedroom units, half of the apartment could be used for quarantining a resident if bunk beds were installed in the other bedroom. Although there is no porch or lobby in front of the apartments, the lobby on the ground floor could implement some contagion-prevention measures. The complex is designed as a group of towers that comprise 77 apartments well integrated into the adjacent downtown Berkeley neighborhood, which is characterized by detached single-family homes, granny flats apartment buildings, a bungalow court, and garages and workshops that have been converted into living units. Two towers stop at the third level, creating communal seating “living rooms” that can, weather permitting, be used throughout the day, providing open-air opportunities for interaction within a safe environment. One level up, one finds roof terraces to grow crops that ensure a secure food supply and provide a space for recreational productive activities (see Figure 10). Research indicates that farming activity is beneficial for calming anxieties triggered by the pandemic.

**Figure 11:**
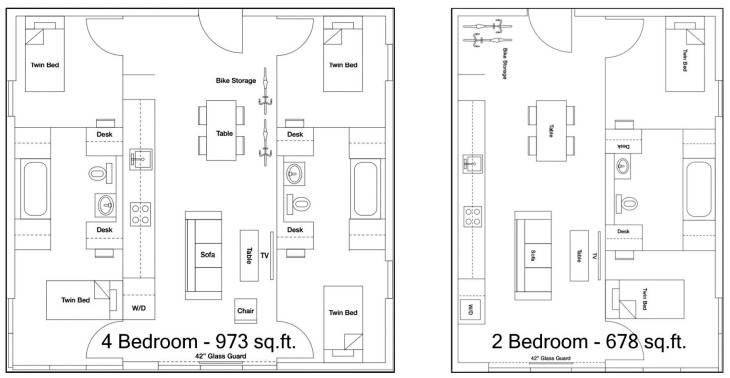
Garden Village Roof Farm (courtesy Natoma Architects Inc.).

The internal arrangement of the units and similar sizes of the bedrooms make the exchange of room functions easy, providing flexibility during a pandemic without adding costs (see Figure 11) (see Table 1). The developer Nautilus Group used prefabricated components for the Garden Village complex bedrooms, featuring two main modules: TYPE A (678 sq ft living/dining/kitchen) and TYPE B (973 sq ft two bedroom/bathroom) (see Figure 11). These modules are manufactured off-site and assembled, similar to the automobile industry. This modular approach reduces waste, enhances quality, and, even accounting for transportation and weather protection, lowers costs by 20%.

## 7. Re-Interpretation of Garden Apartments: Tassafaronga Village

Originally developed in 1945 for war workers, the housing complex was taken over by the Oakland Housing Authority (OHA) in 1955. An 87-unit public housing project was built in 1964 but later became distressed and crime-ridden. The site spans an industrial-residential divide acquired in 2006 by OHA.

**Figure 12:**
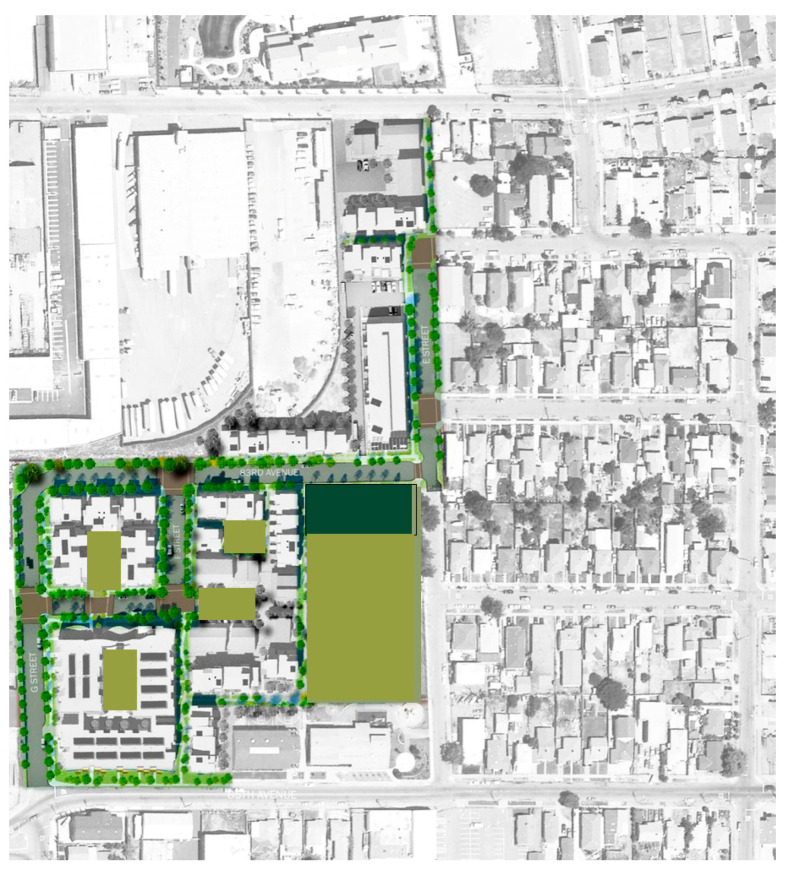
Tessafaronga Village site plan (Image courtesy David Baker Architects, San Francisco, CA, USA).

Additional parcels demolished the outdated housing and created a new seven-acre affordable housing community. Tassafaronga Village now features 60 affordable family rental apartments, 77 affordable rental townhouses, 20 supportive apartments with an in-house clinic space, and 22 integrated Habitat for Humanity townhouses for first-time homeowners (see Figure 12) [13,47,48].

While a large public plaza anchors Tassafaronga Village, each of the three new housing areas also has a semi-private shared space, creating sheltered play and gathering places for residents. New landscaped paths and traffic-calmed roadways connect new housing to the previously isolated school, library, and community center, providing the community with excellent access during a pandemic. In its provision of social access to facilities and the provision of a community center, Tessafaronga Village is most similar to the garden apartments.

Situated on seven and a half acres at the southern end of Oakland, Tessasfaronga Village has a range of green pathways, pocket parks, and open spaces. While a large public plaza anchors the new village, each of the three new housing areas also has a semi-private shared space, creating sheltered play and gathering places for children and grown-up residents. These spaces can relieve stress for the residents, particularly during health emergencies.

A three-story apartment building features affordable rental units in varying configurations built with the Oakland Housing Authority. Apartments flank a hidden parking structure and also enclose a second-level open-air courtyard. On either side of a main plaza, twenty-two family townhomes were built in cooperation with the local chapter of Habitat for Humanity. All buildings on the property incorporate solar power and recycled materials. A defunct pasta factory and parcel of unused industrial land were reclaimed as an artist-oriented series of live-work spaces [49].

**Figure 13:**
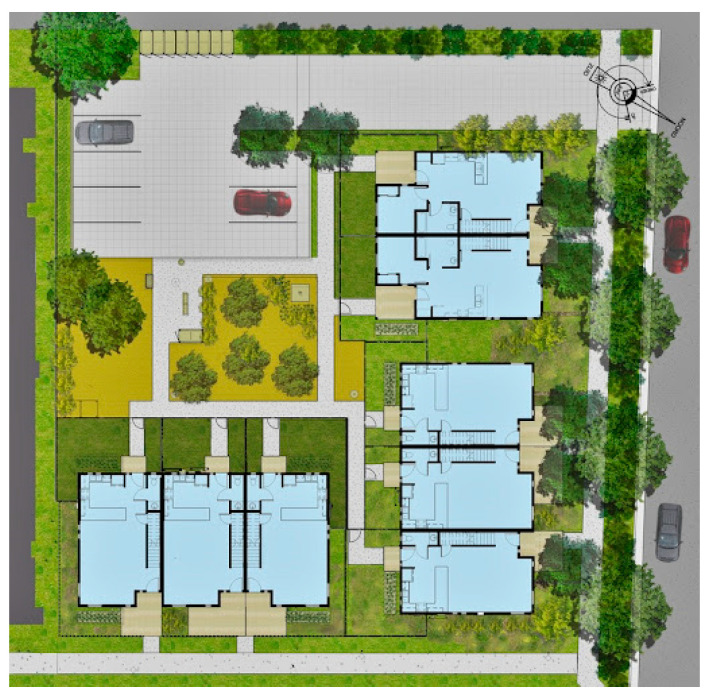
Tassafaronga Village townhouse unit plans (courtesy David Baker Architects).

The complex’s two- and three-story townhouses are distinct from the single-story homes of the surrounding suburban neighborhood, yet they integrate well without causing disruption. Each unit features front and back porches and private garden areas. These spaces serve as a buffer between the indoors and outdoors, eliminating shared entrances that could heighten the risk of contagion during a health emergency. Additionally, they provide a small outdoor area that can be utilized to expand living space if needed during such emergencies, but there is no clear, easy way to set up an office space inside (see Figure 13).

Zoning regulations required the construction of one parking space for each housing unit. A parking structure was built beneath the residential buildings to comply with this requirement, with a courtyard on top. This design allowed for 40% of the 7.5-acre development to be dedicated to landscaping, setting it apart from competitors who typically arrange townhouses around parking lots.

**Figure 14:**
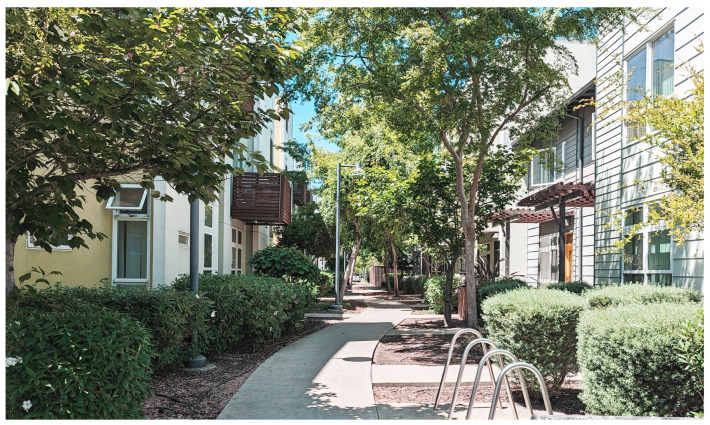
Terssafaronga Alley with townhouses (photo by the author 2023).

The landscape includes drought-tolerant shrubs and trees, which provide shade and reduce the heat island effect, creating a calming environment that helps alleviate anxiety, particularly during a pandemic. (see Figure 14). The chosen plant palettes cleanse stormwater from surrounding streets. Runoff from roof drains is captured and treated by biofiltration planters. All plantings are climate-adapted for low-water use and maintained by drip irrigation.

**Figure 15:**
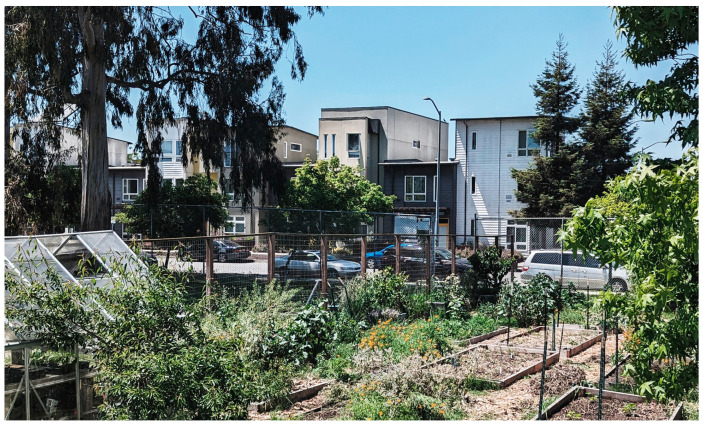
Acta Non-Verba: Youth Urban Farm (Photo by the author).

A vital element of the village is the Acta Non-Verba (ANV) Youth Urban Farm Project, started in 2011 by Navy veteran Kelly Carlisle (see Figure 15). Located in a 0.25-acre garden within a public park, the project offers a safe space for families, promotes healthy living, and addresses food scarcity through a Free Food Pantry and a Community Supported Agriculture (CSA) program. This initiative benefits over 90% of local students who qualify for free or reduced-price lunches. The farm serves as a valuable resource for stress relief, especially if new health challenges arise in the area.

## 8. The Future of California Urbanism

Acknowledging the importance of suburban multifamily housing is crucial for preparing for future pandemics. Casual social interactions are more likely to occur in multifamily developments than in isolated single-family home neighborhoods. These projects can guarantee the provision of green spaces, including space for agricultural activity, playing a vital role in alleviating mental health issues during stressful times, such as a pandemic.

Since 1970, suburban multifamily housing has become the fastest-growing segment of the housing market in the United States, significantly outpacing the growth of suburban single-family homes (US Census Bureau, 1973–2007). Typically, suburban multifamily housing comprises 20 to 30 units per acre and is primarily rental property. It is an existing and effective model for introducing increased density into suburban areas. Additionally, the green spaces associated with multifamily developments can be integrated into the traditional dense, compact city model [50,51]. When near commercial centers, multifamily complexes with green roofs, such as Natoma Architect’s Garden Village, can connect to farming areas over commercial buildings.

As real estate prices in California continue to rise, individuals from various income levels are increasingly moving into townhouses and multifamily complexes. Daybreak Grove is an excellent example of integrating townhouses into a suburban neighborhood without causing disruption. The stigma that used to be associated with suburban multifamily housing—linking it to low-income status, strain on local schools, and a decrease in adjacent property values—is unfounded and is gradually fading away [52].

Cities are unlikely to secure the necessary funds to create sufficient new public green spaces to ensure easy access for all citizens. Therefore, providing green spaces through private developments is the more viable and realistic approach, and it represents a key strategy for the healthy future development of cities in California.

Multifamily developments should be recognized for their potential to provide communal green spaces, opportunities for informal encounters, and venues for physical exercise and growing food. Helping to make these ideas possibly come true are new regulations that have come into effect, speeding the permit process up and allowing new locations for multifamily units. One new California senate bill re-ups and expands a law that speeds up the approval of apartment buildings in which some units are set aside for lower-income Californians, while another bill does something similar for affordable housing on property owned by religious institutions and non-profits, providing flexibility to exceed or override local zoning, greater certainty about the timing and likelihood of planning approvals, and substantial relief from environmental review and litigation [53]. Legislation aiming to curtail the use of the California Environmental Quality Act (CEQA) to stop or delay new housing construction on the basis of new residents’ actions being classified as “pollution” and impactful to the environment was also passed [54].

To fully realize the potential of suburban multifamily housing, we must view these complexes as an integral part of the larger urban landscape rather than as isolated enclaves [55]. Planners must rethink how multifamily housing complexes interact with commercial and residential areas, including single-family homes. This requires a shift in perspective regarding typical commercial strip mall developments, which should not only be designed for cars but also accommodate pedestrian and bicycle connections.

Promoting connectivity and updating zoning regulations accordingly to allow changes but viewing them as housing for all income levels rather than just low-income people is essential. Increasingly, in fact, multifamily residences are attracting higher-income groups, as exemplified by projects like MAD Architects’ Garden House in Los Angeles (see, for example, MAD Architects Garden House in Los Angeles [56]).

In conclusion, developers, urban planners, and landscape designers should prioritize the presence and quality of green spaces within multifamily complexes and visualize them as neighborhood generators. This strategy will foster a healthier environment and enhance the overall quality of life for residents and better prepare them for the next pandemic.

## 9. Conclusions

This study identified three exemplary housing complexes: Daybreak Grove, Tessafaronga Village, and Village Green. These complexes were selected for their diverse locations—hybrid urban/suburban to mixed suburban areas—and green spaces. Each complex features porches, community areas, and thoughtfully designed green spaces that can accommodate a variety of activities beyond their original purposes with minimal economic effort (see Table 1). Furthermore, these complexes support urban farming, enhancing access to fresh food and promoting mental and physical well-being—critical components for effective pandemic preparedness [13,57,58].

U.S. metropolitan areas generally have lower population and employment densities, a more dispersed layout, and a greater reliance on private cars for transportation. This supports the prevalence of single-family neighborhoods. However, multifamily housing complexes, which are very common in Europe, are also becoming increasingly popular in California.

One key difference between housing projects on both sides of the Atlantic is their design. While both may feature patios or recreation areas, European designs often appear less lush, with landscaping frequently intentionally arranged with dry compositions. In contrast, when multifamily housing complexes in the U.S. include patios or gardens, they typically showcase abundant vegetation, likely due to social pressures to conform to a suburban aesthetic. Given vegetation’s benefits for people’s well-being and health, this might be a practice that other countries might want to adopt in a post-pandemic world.

There has been little discussion about the type of housing space required in a post-pandemic world, and there is no strategic thought for post-COVID residential building design. All three case studies presented in this paper can help create new types of neighborhoods by developing a continuously connected network of semi-public green spaces integrated into various environmental settings. This approach would extend the tradition of garden apartments and courtyard living while significantly impacting the health benefits of urban planning in California.

A well-functioning neighborhood should also provide residents with safe and easy access to essential goods and services, such as grocery stores, quality public schools, parks, recreational facilities, civic amenities, and reliable public transit for trips beyond the neighborhood. These neighborhoods should be designed to be walkable and bikeable, featuring interconnected street networks that accommodate people of all ages and abilities.

## 10. Research Limitations

This research involved site visits to various housing projects, but a broader range of case study examples could enhance the understanding of effective spatial strategies to prevent virus transmission in the future. Conducting in-depth interviews with individuals who lived in these complexes during the pandemic’s peak could provide insight into how much the suggested adaptations in spatial configurations were utilized.

The population density in each complex at the time of the pandemic, along with people’s behaviors and cultural or socioeconomic factors, may have influenced the implementation of spatial measures to reduce the risk of virus transmission. Future studies should consider these variables to better characterize effective spatial strategies for preventing transmission.

The case studies examined in this paper showcase positive conditions; however, most existing housing stock faces substantial challenges that negatively affect residents’ quality of life during health emergencies. In some cases, remediation is not feasible, while in others, it is prohibitively expensive. Moreover, these case studies are tailored to their specific locations and circumstances, serving primarily as inspiration for future building projects rather than as exact models to replicate.

## Figures and Tables

**Table 1:** Indicating opportunities for adaptation of the three projects analyzed.

	Daybreak Grove	Garden Village	Tessafaronga
Opportunities for exercising	Y	No	Y
Ability to isolate within the home	Y	Y	No
Opportunities for social interaction	Y	Y	Y
Green Space	Y	Y	Y
Ability to set-up home-office within the home	Y	Y	No
Farming opportunities	Y	Y	Y
Ability to set-up a work station outside	Y	No	Y

## Data Availability

The original contributions presented in the study are included in the article; further inquiries can be directed to the corresponding author.
